# An Improved Inactivated Influenza Vaccine with Enhanced Cross Protection

**DOI:** 10.3389/fimmu.2018.01815

**Published:** 2018-08-09

**Authors:** Yawei Ni, Jianhua Guo, Debra Turner, Ian Tizard

**Affiliations:** ^1^KJ Biosciences LLC, College Station, TX, United States; ^2^Department of Veterinary Pathobiology, Texas A&M University, College Station, TX, United States

**Keywords:** influenza, inactivated vaccine, HA2, low pH, cross protection

## Abstract

Current inactivated influenza vaccines are strain-specific and poorly effective against variant or mismatched viruses. They are standardized based on their hemagglutinin (HA) or ability to induce strain-specific hemagglutination inhibition (HAI) antibodies. The HA is known to undergo major conformational changes when exposed to the low pH environment of endosomes (pH 5.0 and 37°C), which are required for membrane fusion during virus cell entry. In an effort to improve these vaccines, influenza antigens treated under various low pH conditions were evaluated for increased cross-reactive antibody response and cross protection. It was found that a full range of structural and antigenic changes in HA could be induced by varying low pH treatment conditions from the mild (low pH at ≤25°C) to the strong (low pH at ≥37°C) as determined by analysis of potency, HA morphology, protease sensitivity, and reactivity with an anti-HA2 domain (CD) antibody. Inactivated antigens of both H1N1 and H3N2 strains treated at mild low pH conditions (0–25°C) exhibited only moderate HA structural and antigenic changes and markedly increased antibody response against HA2, the highly conserved part of HA, and cross protection against heterologous challenge in mice by up to 30% in survival. By contrast, antigen treated with low pH at 37°C showed more extensive structural and antigenic changes, and induced much less of an increase in antibody response against HA2, but a greater increase with response against HA1, and did not provide any increased cross protection. These results suggest that the increased response against HA2 obtained with the mild low pH treatment is associated with the increased cross protection. These antigens treated at the mild low pH conditions remained capable of inducing a high level of strain-specific HAI antibodies. Thus, they could readily be formulated as an inactivated influenza vaccine which not only provides the same strain-specific protection but also an increased cross protection against heterologous viruses. Such a vaccine could be particularly beneficial in cases of vaccine mismatch.

## Introduction

Current inactivated influenza vaccines, either trivalent or quadrivalent (TIV or QIV; H1N1, H3N2, and one or two B strains), are strain-specific and poorly effective against variant or mismatched viruses. They are only moderately effective against seasonal influenza with an efficacy of 59% in adults aged 18–65 as shown by meta-analysis of clinical studies over several influenza seasons (1). Their effectiveness can decrease significantly with mismatched viruses and be as low as 19% as recently occurred in the 2014–2015 season (2, 3). Such vaccine mismatch still occurs frequently despite an annual vaccine strain exchange intended to match the circulating viruses. It also occurred in the current influenza season (2017–2018) with an interim estimated vaccine effectiveness of 36% (3). This underscores the fact that influenza viruses undergo constant genetic and antigenic changes and highlights the urgent need to develop better vaccines with a broader spectrum of protection (4, 5). Toward this goal, a strategic plan for developing a universal influenza vaccine was recently published by NIAID of NIH (6).

The hemagglutinin (HA) is the protective antigen in inactivated influenza vaccines (7). These vaccines are standardized based on specific amount of HA protein or potency as measured by the single radial diffusion (SRD) assay (8). They induce strain-specific antibody responses as measured by hemagglutination inhibition (HAI) and neutralization. A HAI titer of 1:40 is considered protective in humans. The HA is a trimeric protein made of three identical subunits. Each subunit consists of two parts, HA1 and HA2. The HA1 constitutes the globular head of HA and is the major target of immune responses. It contains the cell receptor binding site and is therefore the basis for hemagglutination activity and HAI assay. It is highly variable, which is the primary cause of vaccine mismatch. On the other hand, HA2 makes up the bulk of HA stem and is less immunogenic, but highly conserved within subtype or closely related subtypes (9). It consists of several distinct domains including fusion peptide and C and D helixes or the long alpha helix (LAH) (10). During cell entry, the virus is taken up into endosomes following receptor binding where the HA is exposed to the low pH environment within the endosome (~pH 5 at 37°C). Under this low pH condition, HA undergoes drastic conformational changes including dissociation of the HA1 globular head and refolding and rising of the HA2 (11, 12). These conformational changes facilitate the membrane fusion and subsequent delivery of the viral genome into the cell cytoplasm.

The HA2, being highly conserved, has been a major target for the development of a broad-spectrum or universal influenza vaccine. It forms the central part of HA stem. The current inactivated vaccines induce only a minimal level of anti-HA2 or HA stem antibodies (13). This is consistent with HA stem being masked by the HA1 globular head. However, antibodies induced against the HA stem, either neutralizing or non-neutralizing, can be broadly protective (13–16). The non-neutralizing antibodies against conserved domains have been increasingly recognized to play a significant role in cross protection *via* the antibody-dependent cytotoxicity (ADCC) (15–17). Various approaches are being evaluated to enhance immune responses against HA2 or HA stem, including chimeric HA (18), HA stem constructs (19–21), and selected HA2 domains including the CD or LAH (22, 23). Based on exposure of the HA2 at low pH, inactivated antigens treated at pH 5.0 and 37°C have also been evaluated, but found to be incapable of inducing any increased cross-reactive antibody responses or increased cross protection (24).

To further evaluate the potential of low pH-treated antigens for increased cross-reactive immune response and cross protection, we treated the antigens in a wide range of low pH conditions, characterizes them based on different antigenic and structural parameters (antigenicity, morphology, protease sensitivity and reactivity with an anti-CD antibody), and evaluated the antigens generated under different low pH conditions for immunogenicity and protection in mice. It was found that it was the antigens treated under the mild low pH conditions (at ≤25°C), but not that treated at the strong low pH condition (37°C) that could induce increased antibody response against HA2 and cross protection. These antigens exhibited only moderate structural changes in HA as compared to the one generated at the strong low pH condition (37°C). These results together potentially form the basis for an improved inactivated influenza vaccine with increased cross protection.

## Materials and Methods

### Antigens

The influenza antigens used in present studies included (1) inactivated whole virus (IWV) antigens of A/New Caledonia/20/99 (H1H1 NC), A/Puerto Rico/8/34 (H1N1 PR8), A/Victoria/361/2011 (H3N2 Vic), and A/Hong Kong/1/1968 (H3N2 HK), (2) inactivated influenza vaccines: TIV 2011–2012 [Flulaval; containing A/California/7/09 (H1N1), A/Victoria/210/2009 (H3N2), and B/Brisbane/60/2008], monovalent H1N1 CA (A/California/07/2009) and monovalent H5N1 VN (A/Vietnam/1203/04) vaccine, and (3) recombinant HAs of H1N1 NC, H1N1 PR8, H1N1 CA, H3N2 Vic, H3N2 HK, H2N2 SG (A/Singapore/1/57), and H5N1 VN (BEI resources, NIAID, NIH and Sino Biologicals, Inc.). The IWV antigens were prepared by purification from infected MDCK cells and inactivation with formaldehyde as described previously (25, 26). Inactivation was performed by keeping virus preparations in 0.01% formaldehyde at 4°C for at least 3 days. Viruses were then pelleted to remove formaldehyde, re-suspended in phosphate-buffered saline (PBS; 20 mM phosphate, 150 mM NaCl, pH 7.4) and stored at 4°C prior to use. The antigen protein concentrations were determined by bicinchoninic acid (BCA) assay (Thermofisher Scientific).

### Low pH Treatment

For low pH treatment, the pH of antigen solutions in PBS was adjusted to different levels (4.8–6.2 in 0.2 increments) with low pH citric-phosphate (Na_2_HPO_4_) buffers (pH 4.4–5.4 in 0.2 increments; 0.2 M) or dilute HCl (0.1 M). The use of the same low pH buffers with incremental difference in pH was to ensure the consistent buffer concentration in treated antigen preparation and the better control of different pHs over the wide range used. The antigens were then incubated at 0°C (on ice), 4°C, 25°C [room temperature (RT)], or 37°C for 15 min before the pH was adjusted to ~7.0 with 1 M pH 8.0 citric-phosphate buffer. Trial runs were performed before each experiment to ensure the correct pH was reached following the pH adjustment. The treated antigens were stored at 4°C prior to use. In the case of purified live viruses, formaldehyde was added to inactivate the viruses as described above immediately after low pH treatment. The viruses were then pelleted and suspended in PBS and stored at 4°C prior to use.

### Potency

The potency of inactivated antigens or the amount of HA protein was measured by the SRD test as described previously (8) using the anti-HA reference sera and reference antigens (H1N1 NC and H3N2 Vic) obtained from Center of Biologics Evaluation and Research of the FDA. The test and reference antigens were used at appropriate concentrations according to the reagent use instructions. Each dilution of the test and reference antigens was tested in duplicate and the diameter of the precipitation ring was measured at both horizontal and vertical directions to generate the mean diameter. The HA concentration (μg/ml) in the test antigens was calculated based on the mean precipitation ring sizes (diameters) by linear regression with the reference antigens as the standards.

### Transmission Electron Microscopy (TEM)

Transmission electron microscopy was performed using the sodium phosphotungstate negative stain to examine morphology of virus particles at the Imaging Center of College of Veterinary Medicine and Biomedical Sciences, Texas A&M University.

### Immunization and Challenge

All animal studies were conducted with the approval of the Institutional Animal Use and Care Committee at Texas A&M University. 6- to 8-week-old female Balb/c mice (*n* = 5–7) were immunized with the treated or untreated IWV or vaccine antigens in 50 µl by intramuscular injection twice, 4 weeks apart. The antigen dose was 5 µg total protein or 1 µg HA/mouse. Serum samples were collected every 2 weeks until 2 weeks after the second immunization. Challenge was performed at week 3 after the second immunization by intranasal delivery of a lethal dose of H1N1 PR8 (1 × 10^6^ TCID_50_) or H3N2 HK (1 × 10^5^ TCID_50_) in 50 µl. During challenge, mice were briefly sedated with isoflurane. After challenge, body weight and survival were monitored daily for 14 days.

### ELISA

Indirect ELISA was used to measure serum-specific antibodies. The 96-well Maxisorp plates (NUNC, Inc., Naperville, IL, USA) were coated overnight at 4°C with IWV (5 µg/ml) or recombinant HA (1 µg/ml) in 50 mM carbonate buffer (pH 9.6) (100 μl/well). Plates were washed three times with PBS-T buffer (PBS with 0.025% Tween 20 and 0.02% NaN_3_) and then blocked for 2 h with PBS-T containing 3% BSA (Sigma Chemical Co.). Serum samples were serially diluted in the same buffer with 3% BSA and incubated at RT for 2 h at RT. Following washing as described above, affinity-purified alkaline phosphatase-conjugated anti-mouse IgG whole molecule (Sigma Chemical Co.) were added at 1:5,000 dilution (100 μl/well) and incubated at RT for 1 h. After wash, each well received 100 µl of substrate p-nitrophenylphosphate prepared according to the instructions from the manufacturer (Pierce, Rockford, IL, USA). The reaction was stopped after a 30-min incubation by addition of 50 µl of 2 M NaOH. Optical density at 405 nm was then measured.

For measurement of low pH-induced structural changes in HA, an ELISA (anti-CD ELISA) was performed with a mouse anti-CD polyclonal antibody (serum) which was generated with the CD domain of H1 subtype consensus sequence fused to a nanoparticle carrier (EcDps, DNA-binding protein from starved cells of bacteria from *E. coli*) (23). Untreated and low pH-treated antigens were coated onto plates as described above. Alternatively, untreated antigens were coated onto plates followed by on-plate treatment at various pH with 20 mM citric-phosphate buffer in saline. The rest of ELISA steps were performed as described above. The anti-CD antibody was used at a dilution of 1:2,000 or higher.

### Hemagglutination Inhibition

Hemagglutination inhibition was performed using chicken red blood cells as described in the WHO manual on animal influenza diagnosis and surveillance (27). Briefly, pooled serum samples were treated with receptor destroying enzyme at 37°C overnight and then inactivated at 56°C for 30 min before being used in HAI test with fresh chicken red blood cells. The HAI titer was the highest dilution with complete inhibition of agglutination (streaming down of RBC dots when the plate was tilted).

### Neutralization Test (NT)

Neutralization test was performed with MDCK cells in 96-well plates. Pooled serum samples from each group were inactivated at 56°C for 30 min and serially diluted twofold before mixing with 100 TCID_50_ virus in duplicate. After incubation at 37°C for 1 h, the mixtures were transferred to MDCK cells and incubated at 37°C for 1 h. The plates were then washed twice with PBS after removing the mixtures, and fresh serum-free media containing 1 µg/ml trypsin was added. The plates were incubated at 37°C for 72 h and then fixed in formalin and stained by crystal violet. The neutralization titer was considered the highest dilution with an intact cell monolayer.

### Immunoblot

Antigen proteins were separated by SDS-PAGE and blotted onto Immobilon P polyvinylidene difluoride membranes (Millipore). Blotted membranes were first blocked at RT in PBS-T buffer containing 3% BSA for 2 h. They were then probed at RT for 2 h with the pooled serum sample diluted at 1:2,000 or higher in the same buffer with BSA. After wash with PBS-T, they were incubated at RT for 1 h with the alkaline phosphatase-conjugated anti-mouse IgG Fc (Sigma Chemical Co., St. Louis, MO, USA). Membranes were developed with substrate BCIP (5-bromo-4-chloro-3-indolylphosphate)–NBT (nitroblue tetrazolium) (Sigma Chemical Co.) for 30 min. Densitometry was performed using NIH ImageJ (http://rsbweb.nih.gov/ij/) for comparing the optical densities of positive bands on the same blot. For each blot, the density measurement was performed twice with the mean value presented.

### Statistics

Each serum sample was tested in duplicate with mean value presented. Each immunoblot test was repeated at least once. The densitometry measurement for each immunoblot was performed twice with the mean value presented. The mean body weight was determined for each animal group. Linear regression was used to calculate protein and antigen concentrations for BCA and SRD assays. Means were compared using the Student’s *t*-test. The increased survival by low pH-treated antigen was analyzed against the untreated antigen by Chi-square goodness of fit test. A *p* value ≤0.05 is considered significant.

## Results

### Induction of Antigenic and Structural Changes in HA in Correlation With Low pH Treatment Conditions

Inactivated influenza antigens, vaccines, or purified live viruses were treated at low pH under various conditions and then evaluated for antigenic changes by potency and for structural changes by electron microscopy (EM), protease sensitivity, and reactivity with a polyclonal antibody specific to the CD domain of HA2. In the case of live viruses, viruses were subjected to inactivation immediately after the low pH treatment as described in Section “Materials and Methods.”

#### Antigenic Changes

**Figure 1:**
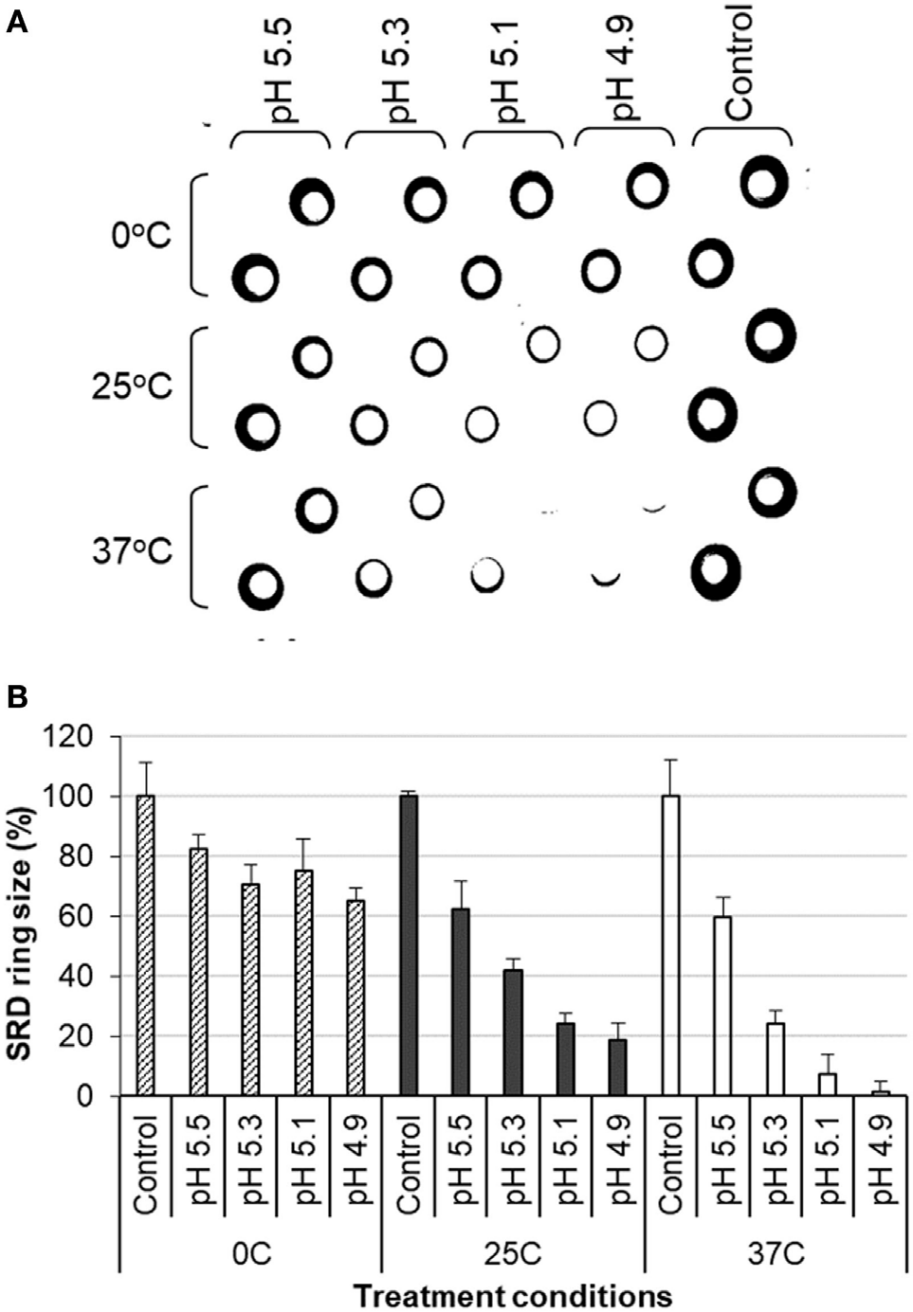
Analysis of influenza inactivated whole virus antigen (H1N1 NC) by single radial diffusion (SRD) following low pH treatment. The low pH treatment and SRD were performed as described in Section “Materials and Methods.” **(A)** SRD plate. **(B)** Percent of precipitation ring sizes as compared to the untreated control.

The antigenic changes of the treated antigens were measured by the SRD assay which was conducted using reference polyclonal anti-HA antibodies for individual vaccine strains. It was found that the potency of inactivated antigens decreased in direct correlation with an increase in the stringency of the low pH treatment conditions. The reduction in potency was shown by reduction in the size of the precipitation ring on the SRD plate (Figures 1A,B). Higher temperatures and lower pH led to a greater loss of potency (Figures 1A,B). Increasing temperature drastically enhanced the effect of low pH (Figures 1A,B). The results from one experiment with H1N1 NC IWV antigens treated at pH 5.2 and different temperatures are shown in Table 1. A full range of potency loss (10–90%) could be generated by varying the temperature from 0 to 37°C at the low pH used (Table 1).

**Table 1:** Potency and immunogenicity of H1N1 NC inactivated whole virus antigens treated at pH 5.2 and different temperatures.

Groups	Potency [hemagglutinin μg/ml (% reduction)]	ELISA		Hemagglutination inhibition		Neutralization test	
		H1N1 NC	H1N1 PR8	H1N1 NC	H1N1 PR8	H1N1 NC	H1N1 PR8
Control	133.4 (0%)	128,000	8,000	320	<10	2,560	<20
0°C	116.7 (12.5%)	128,000	16,000	320	<10	2,560	<20
25°C	72.74 (45.5%)	256,000	32,000	160	10	2,560	20
37°C	13.3 (90.0%)	256,000	32,000	160	<10	1,280	<20

#### Structural Changes

##### Protease Sensitivity

**Figure 2:**
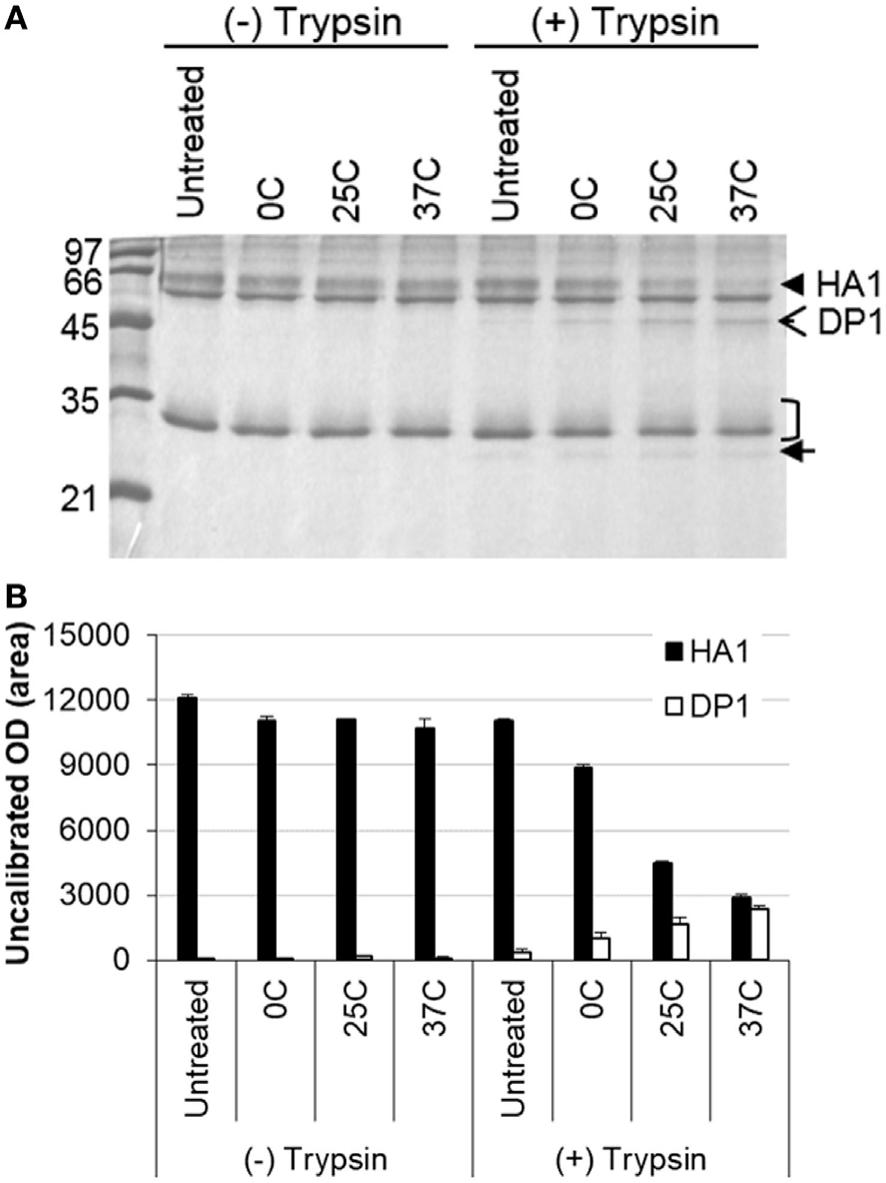
Analysis of influenza inactivated whole virus antigen (H1N1 NC) by protease digestion following low pH treatment. The antigen was treated at pH 5.2 and different temperatures (0, 25, and 37°C) and then subjected to digestion with trypsin. **(A)** SDS-PAGE gel stained with Coomassie blue. **(B)** Densitometry analysis of protein bands in the SDS-PAGE gel. The density measurement was performed twice and mean values are presented. The closed arrowhead indicates the HA1, open arrowhead indicates the HA1 digestion product (DP1), the bracket indicates the HA2/M1, and closed arrow indicates the trypsin.

It is known that structural changes of HA induced at the low pH render it sensitive to the protease digestion (11, 12). Thus, IWV antigens (H1N1 NC) treated at different low pH conditions were analyzed for protease (trypsin) sensitivity. The results showed that HA1 in the treated antigens was gradually digested along with increase in the stringency of low pH treatment conditions (pH 5.2 and 0–37°C) (Figures 2A,B). The HA1 in the antigen treated at 37°C disappeared almost completely, whereas most of the HA1 in the antigen treated at 0°C remained intact. The gradual digestion of HA1 and associated structural changes therefore correlated with the treatment conditions (Figures 2A,B). The effect on the HA2 could not be observed as it co-migrated with the M1 protein.

##### Electron Microscopy

**Figure 3:**
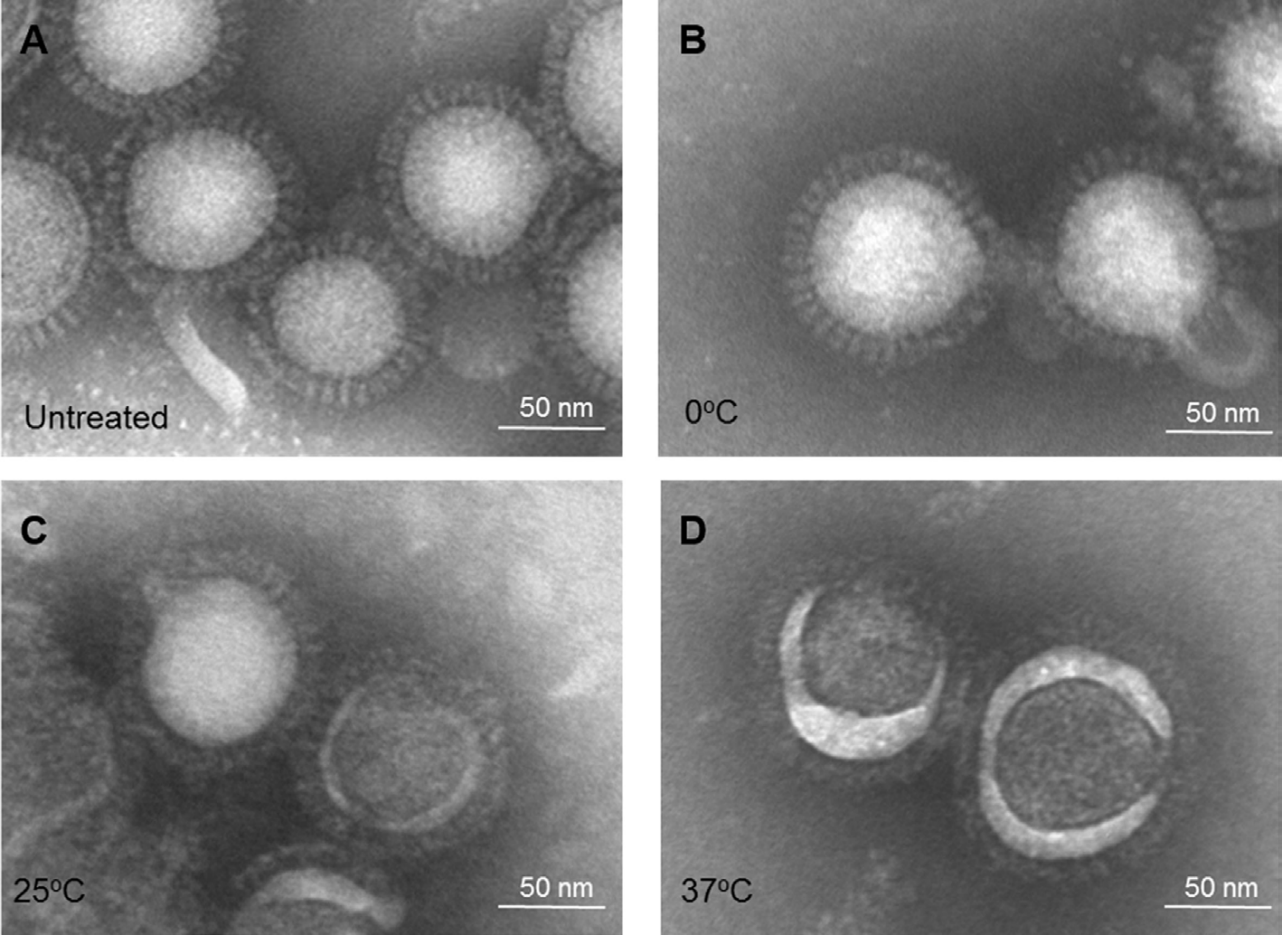
Electron microscopy of influenza inactivated whole virus antigen (H1N1 NC) treated at pH 5.2 and different temperatures. **(A)** Untreated control. **(B)** Treated at 0°C. **(C)** Treated at 25°C. **(D)** Treated at 37°C.

The same treated IWV antigens (H1N1 NC) were then examined for morphological changes by EM. The HA spike in the antigen treated at pH 5.2 and 0°C exhibited no apparent change as compared to the untreated control (Figures 3A,B). However, antigen treated at pH 5.2 and 25°C appeared to be less well defined, but retained the overall spike appearance (Figure 3C). The HA spike structure was totally lost in antigens treated at pH 5.2 and 37°C with the HA spikes collapsing into an amorphous mass on the surface of virus particles (Figure 3D). These results showed that the change in HA spike morphology also increased in association with increase in the stringency of low pH treatment conditions (from 0 to 37°C).

##### Reaction With Anti-CD Domain Antibody

The CD or LAH (aa 76–130) of HA2 forms the central part of HA stem. It is partially hidden in the normal HA conformation, but refolded and fully exposed when exposed to the low pH environment within endosomes (~pH 5.0 and 37°C) (11, 12). To further characterize low pH-induced HA structural changes, an anti-CD polyclonal antibody was used to react with treated influenza antigens. This specific antibody was generated in mice using the CD domain (H1 consensus) fused to a nanoparticle carrier protein (23). It has been shown to be protective against lethal challenge (23), thus confirming its ability in recognizing corresponding domains in native antigens.

**Figure 4:**
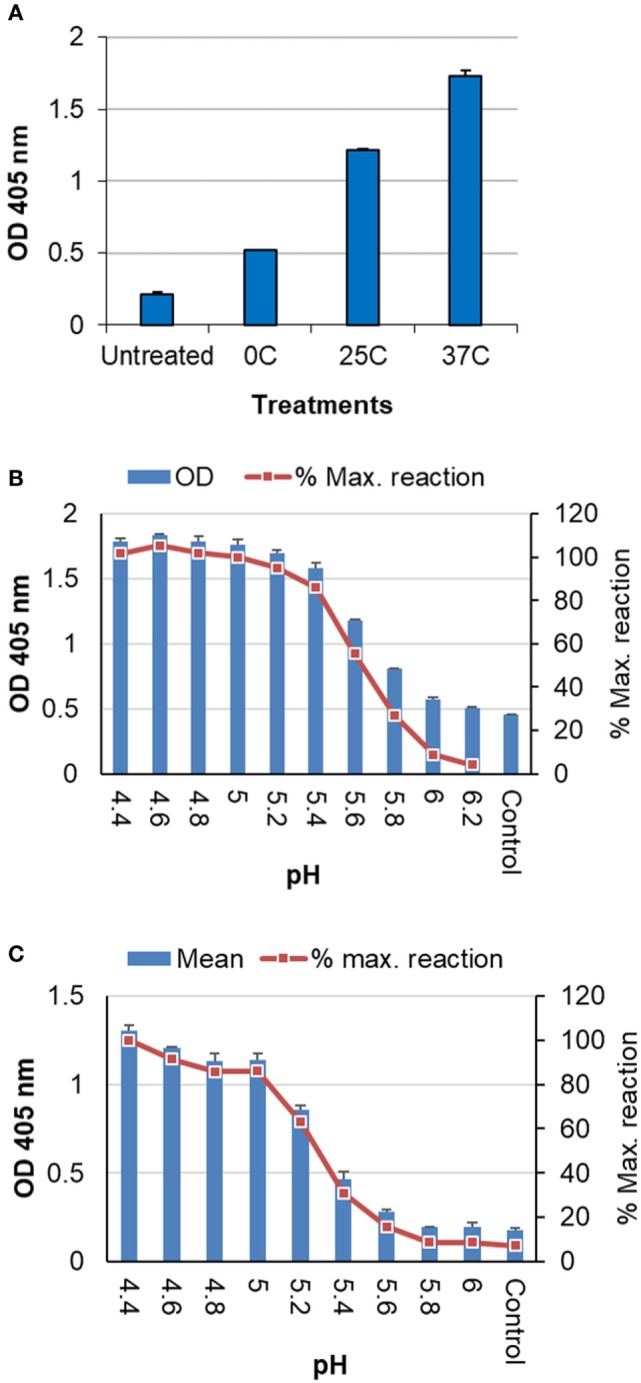
Analysis of inactivated influenza antigens by anti-CD ELISA following low pH treatment. **(A)** Inactivated whole virus (IWV) antigens (H1N1 NC) treated at pH 5.2 and different temperatures (0, 25, and 37°C) were used to coat the plate for ELISA. **(B)** Untreated IWV antigen (H1N1 NC) was coated onto plate followed by on-plate treatment at different pHs and 37°C before wash and the blocking step for ELISA. **(C)** Untreated TIV (2011–2012) was coated onto plate as antigen followed by on-plate treatment at different pHs and 37°C before wash and the blocking step for ELISA. The anti-CD antibody was used at a dilution of ≥1:2,000.

Analysis by ELISA showed that antigens treated at different low pH conditions as listed in Table 1 exhibited an increasing level of reaction with this antibody along with increase in the stringency of low pH treatment conditions (from 0 to 37°C) (Figure 4A), indicating the increased exposure of HA2 after low pH treatment. The antibody was used at a high dilution (≥2,000) to limit the reaction level with the control or untreated antigens. A ~2-fold increase in the reaction was observed with the antigen treated at 0°C as compared to the untreated antigen, while the highest level of reaction was obtained with antigen treated at 37°C (>4-fold) (Figure 4A).

Further analysis showed that a full range of the reaction with this anti-CD antibody could be obtained by gradually decreasing the pH at 37°C till the maximum level of reaction (≤pH 5.0) was reached. The maximum level of reaction likely represented the full CD exposure or HA structural changes (11, 12). Thus, extent of the structural changes at various different conditions can be assessed against this maximum level of reaction for a given antigen (% maximum reaction). These results were obtained with both H1N1 NC IWV (Figure 4B) and a TIV (2011–2012) which contains the 2009 H1N1 strain (Figure 4C). In addition, this analysis could be performed with antigens coated on the plate and on-plate low pH treatment (Figures 4B,C), suggesting that this anti-CD ELISA could be a simple assay for evaluation of the low pH sensitivity of influenza antigens in a quick manner.

Together, these results showed that antigenic and structural changes in HA as measured by different parameters correlate well with each other under different low pH treatment conditions and a full range of such changes in HA can be induced in direct correlation with low pH treatment conditions.

### Induction of Increased Cross-Reactive Antibody Responses by Low pH-Treated Inactivated Antigens

The low pH-treated antigens generated in Table 1 were used to immunize mice for analysis of antibody responses as described in Section “Materials and Methods.”

#### HAI and NT Antibodies

The pooled serum samples were tested by HAI and NT using both homologous and heterologous viruses. The treated antigens induced lower HAI and NT titers against the homologous virus (H1N1 NC) which correlated with reduction in their potency (Table 1). However, antigens treated at mild conditions (0–25°C) with a partial potency loss (12–45%) could still induce high levels of HAI and NT antibodies that were either the same or only twofold lower as compared to the untreated antigen (Table 1). Neither untreated nor treated antigens induced any detectable HAI or NT antibodies against the heterologous H1N1 PR8 virus (A/Puerto Rico/8/34), except for one treated at 25°C at the lowest dilution (1:10 or 1:20) (Table 1).

#### Increased Cross-Reactive Antibody Responses as Measured by ELISA

**Figure 5:**
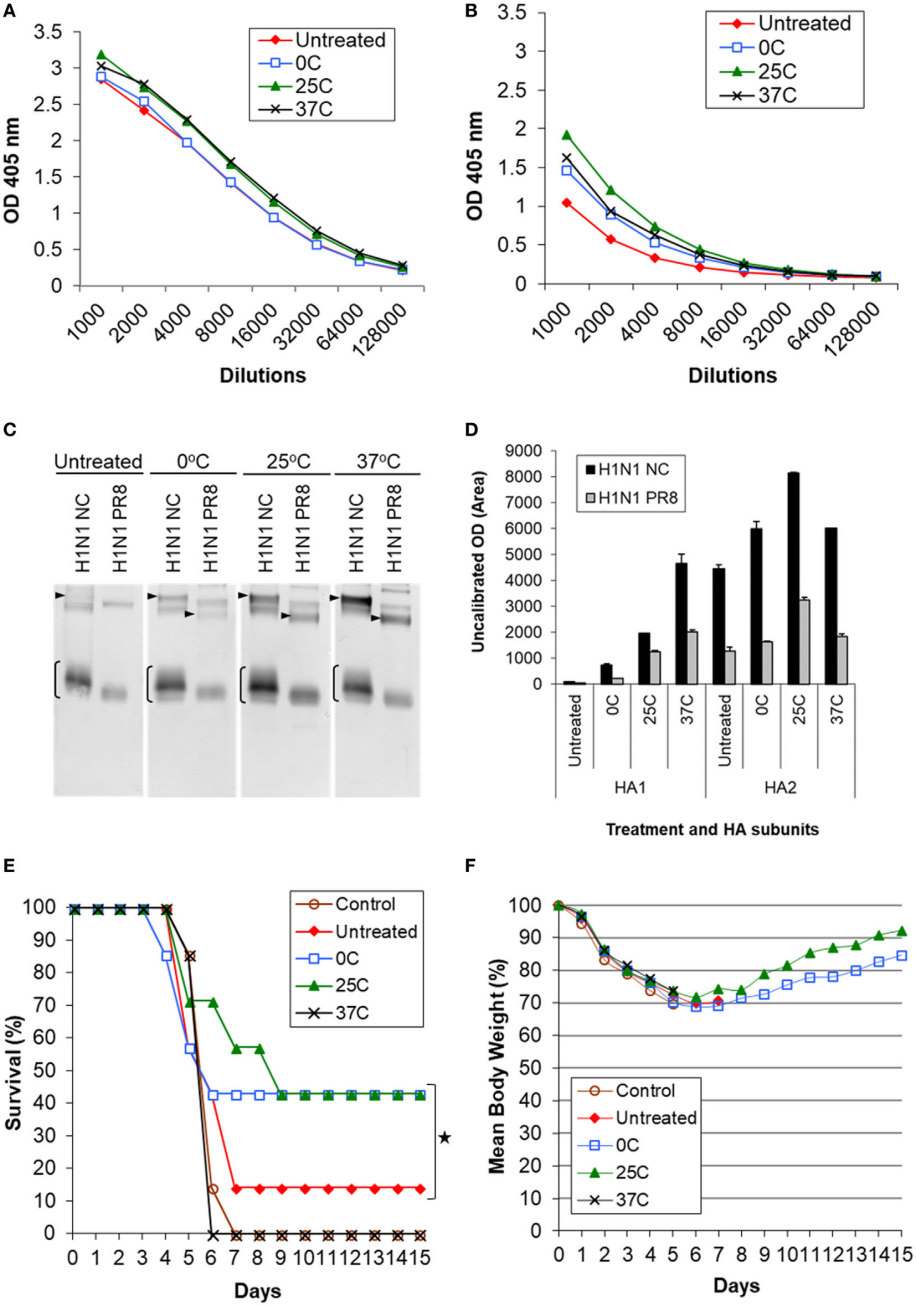
Induction of cross-reactive antibody response and cross protection by low pH-treated inactivated whole virus (IWV) antigens (H1N1 NC) in mice. Mice (*n* = 7) were immunized twice, 4 weeks apart, with IWV antigen treated at pH 5.2 and different temperatures (0, 25, or 37°C). Pooled serum samples were obtained at week 2 after the second immunization and tested for cross-reactive antibody response. Mice were challenged with a highly lethal dose of heterologous H1N1 PR8 virus at week 3 after the second immunization. **(A)** ELISA with the homologous rHA antigen (H1N1 NC), **(B)** ELISA with the heterologous rHA antigen (H1N1 PR8). **(C)** Immunoblot with the homologous (H1N1 NC) and heterologous (H1N1 PR8) IWV antigens. **(D)** Densitometry analysis of positive HA1 and HA2 bands in immunoblot. The density measurement was performed twice for each blot and mean values are presented. **(E)** Survival following challenge. **(F)** Body weight following challenge. Arrowheads indicate HA1. Brackets indicate HA2. Symbol * indicates significant differences (*p* < 0.05).

The pooled serum from each group of mice was tested against homologous and heterologous rHA antigens by ELISA. Both untreated and treated antigens induced high levels of specific antibodies against the homologous antigen (H1N1 NC) (Figure 5A). The antigens treated at 25 and 37°C induced titers twice that induced by the untreated antigen or that treated at 0°C (Table 1; Figure 5A). This indicates that the treated antigens remained highly immunogenic. When tested against heterologous antigen (H1N1 PR8), the overall reaction levels were much lower than those with the homologous antigen (Table 1; Figure 5B). However, all three treated antigens induced increased cross reactivity with the heterologous antigen with a larger increase (fourfold) shown by antigens treated at 25 and 37°C (Table 1; Figure 5B).

#### Increased Cross-Reaction With HA2 as Measured by Immunoblot

The pooled sera were tested by immunoblot using both homologous (H1N1 NC) and heterologous (H1N1 PR8) IWV antigens (Figure 5C). It was found that all treated antigens induced greater antibody response against HA2 as compared to the untreated antigens. Antigens treated at 25°C demonstrated the largest increase in reaction with HA2 (Figure 5C). This was ≥2-fold higher as compared to the untreated antigen as determined by densitometry analysis (Figure 5D). On the other hand, antigen treated at 37°C generated the largest increase in reaction with HA1 (≥4-fold) although it also induced an increased reaction with HA2 (Figures 5C,D). This suggests that the antigenic or structural changes are not only quantitative but also qualitative under different low pH treatment conditions. Importantly, the increased reaction with HA2 also occurred with the heterologous antigen (H1N1 PR8) (Figure 5C). Together, these results suggested that antigens treated at low pH can induce increased antibody responses against HA2 of both homologous and heterologous strains, but a greater level of such antibody response could be obtained with those treated at mild low pH conditions (0 and 25°C) as compared to the strong low pH condition (37°C) (Figures 5C,D; Table 1). As shown above by antigenic and structural analysis, antigens treated at the mild conditions exhibited only limited or moderate structural changes in HA, whereas those treated at the strong condition exhibited the maximum level of such changes (Figures 1–4). This suggests that the extent of structural changes in HA is important in inducing increased response against HA2.

### Increased Cross Protection Against Heterologous Challenge by Inactivated Antigens Treated Under the Mild Low pH Conditions

Mice immunized with antigens listed in Table 1 were challenged with the heterologous H1N1 PR8 virus at a highly lethal dose (1 × 10^6^ TCID_50_). The challenge virus (H1N1 PR8) represented a mismatched virus to the antigen (H1N1 NC) as it exhibited no detectable HAI or NT titer with the latter (Table 1). Antigens treated at the mild conditions (0 and 25°C) exhibited a significantly higher cross protection based on survival (43%) as compared to the untreated antigen (14%) (*p* < 0.05) (Figures 5E,F). By contrast, no detectable increased protection was obtained with the antigen treated at 37°C (Figures 5E,F). Antigen treated at 25°C provided the greatest cross protection as mice in this group survived longer or recovered body weight faster, reaching >90% of the original body weight by the end of the 2-week observation period (Figure 5F). As this antigen induced the greatest reaction with HA2, the observed increased cross protection is apparently associated with moderate structural changes in HA and increased cross reactivity with HA2 obtained at the mild low pH conditions.

### Increased Cross Protection by Mild Low pH Treatment With H3N2 Antigen

We then generated and evaluated H3N2 IWV (A/Victoria/361/2011) treated at mild low pH conditions for increased cross protection. The low pH treatment was performed at two different pHs (5.2 and 5.6) and at 0°C with purified live viruses followed immediately by inactivation with formaldehyde as described in Section “Materials and Methods.” The antigen treated at pH 5.2 lost potency by 55.3% whereas antigen treated at pH 5.6 experienced no apparent loss of potency. It was generally difficult to discern differences within 5% by SRD under the conditions used.

**Figure 6:**
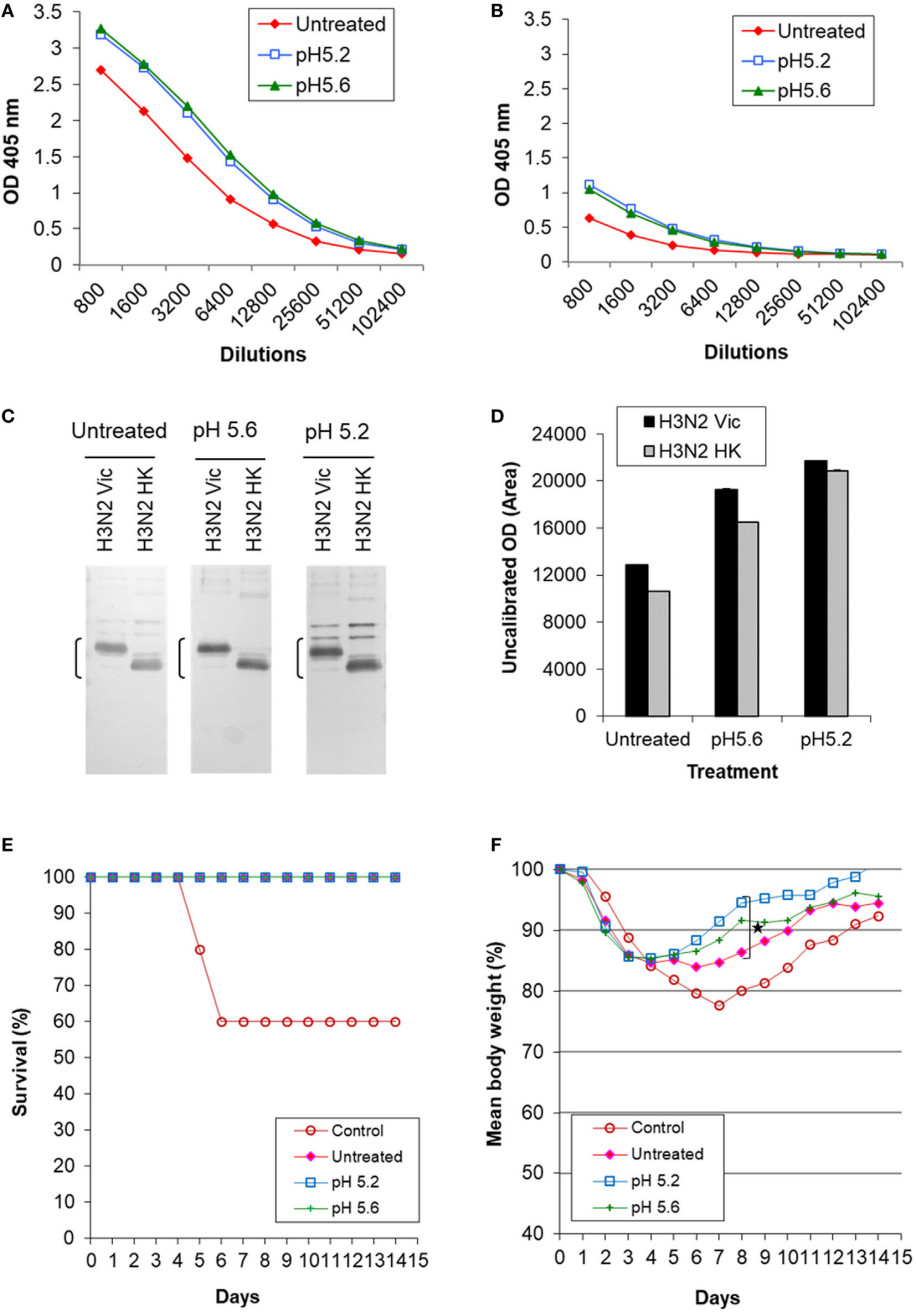
Induction of cross-reactive antibody response and cross protection by low pH-treated inactivated whole virus (IWV) antigens (H3N2 Vic). Mice (*n* = 5) were immunized twice, 4 weeks apart, with inactivated WV antigen (H3N2 Vic) treated at pH 5.2 or 5.6 and at 0°C. Pooled serum samples were obtained at week 2 after the second immunization and tested for cross-reactive antibody response. Mice were challenged with the heterologous H3N2 HK virus at week 3 after the second immunization. **(A)** ELISA with the homologous IWV antigen (H3N2 Vic). **(B)** ELISA with the heterologous IWV antigen (H3N2 HK). **(C)** Immunoblot with the homologous (H3N2 Vic) and heterologous (H3N2 HK) IWV antigens. **(D)** Densitometry analysis of positive HA2 bands in immunoblot. The density measurement was performed twice for each blot and mean values are presented. **(E)** survival following challenge. **(F)** Body weight following challenge. Brackets indicate HA2. Symbol * indicates significant differences (*p* < 0.05).

Both treated antigens induced the same HAI titers as the untreated control against the homologous virus (H3N2 Vic) (Table 2), again indicating that antigens treated at mild conditions remain capable of inducing a high level of HAI antibodies. None of the antigens induced a detectable HAI titer against the heterologous virus (H3N2 HK) as expected (Table 2). As measured by ELISA, the treated antigens induced a twofold higher antibody response than the untreated control against the homologous IWV antigen (H3N2 Vic) (Table 2; Figure 6A), again suggesting that they remain highly immunogenic. Importantly, they induced a much larger increase (fourfold) in response against the heterologous IWV antigen (H3N2 HK) although their overall response levels against the heterologous antigen (H3N2 HK) were much lower than to the homologous antigen (H3N2 Vic) (Table 2; Figure 6B). By immunoblot, the treated antigens also induced increased reaction with HA2. The highest reaction was obtained with the antigen treated at pH 5.2 (Figure 6C) which was nearly twofold higher as compared to the untreated antigen as determined by densitometry analysis (Figure 6D).

**Table 2:** Potency and immunogenicity of H3N2 Vic inactivated whole virus antigens treated under different low pH conditions.

Groups	Potency [hemagglutinin μg/ml (% reduction)]	ELISA		Hemagglutination inhibition	
		H3N2 Vic	H3N2 HK	H3N2 Vic	H3N2 HK
Untreated	23.57 (0%)	51,200	3,200	160	<20
pH 5.2	13.09 (55.6%)	102,400	12,800	160	<20
pH 5.6	24.80 (+5%)	102,400	12,800	160	<20

Mice were challenged with the heterologous H3N2 HK virus which represented a mismatched virus to the antigen (H3N2 Vic) as it exhibited no detectable HAI titer with the latter (Table 2). All immunized mice survived while 60% of the unimmunized control mice died (Figure 6E). However, mice immunized with treated antigens experienced much less loss and faster recovery of body weight as compared to those receiving the untreated antigen (Figure 6F). The antigen treated at pH 5.2 generated least weight loss and fastest recovery with significant difference observed at day 8 as compared to mice receiving the untreated antigen (*p* ≤ 0.05, Student’s *t*-test) (Figure 6F). These results again showed that antigens treated at mild low pH conditions with a moderate potency loss can induce increased antibody response against HA2 and cross protection.

### Further Evaluation of Cross-Reaction With HA2 Induced by Antigens Treated at the Mild Low pH Conditions

HA2 is highly conserved within a subtype and closely related subtypes based on previous studies (9) which are supported by our own sequence alignment analysis (data not shown). Thus, additional antigens of different subtypes were analyzed by immunoblot to further evaluate the cross-reaction with HA2 induced by antigens treated at the mild low pH conditions. The antibodies against the treated antigen were similarly generated with a H1N1 NC IWV treated at pH 5.1 and 0°C with a 21.1% potency reduction in mice (*n* = 5) as described in Section “Materials and Methods,” but without challenge and used as a pooled serum sample. Inactivated antigens used included H1N1 CA and H5N1 VN monovalent inactivated vaccines.

**Figure 7:**
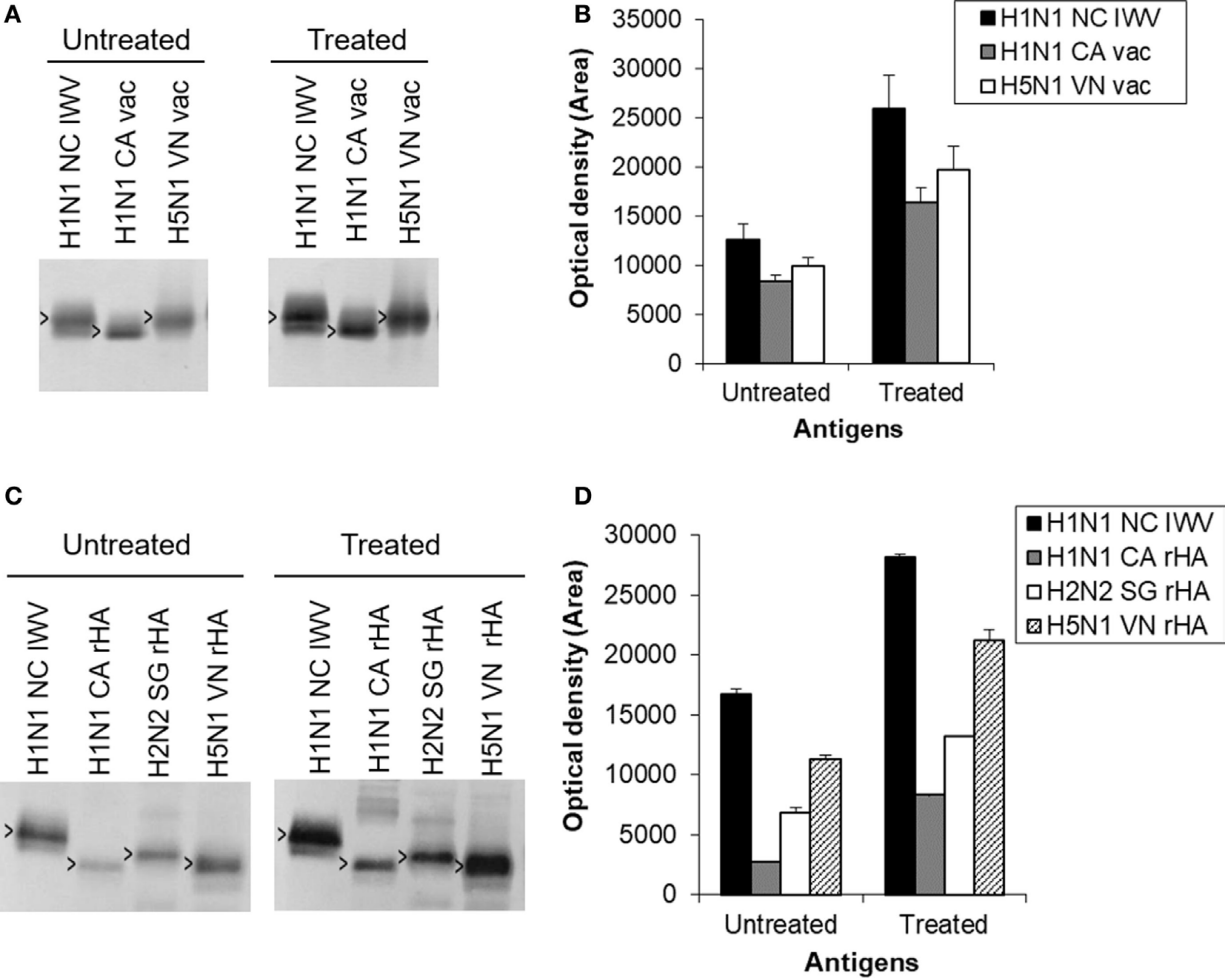
Further evaluation of cross-reaction with HA2 induced by antigens treated at the mild low pH conditions. The antibodies against the antigen treated at the mild low pH condition were similarly generated with a H1N1 NC inactivated whole virus (IWV) treated at pH 5.1 and 0°C with a 21.1% potency reduction in mice (*n* = 5) as described in Section “Materials and Methods,” but without challenge and used as a pooled serum sample. **(A)** Immunoblot with H1N1 NC IWV and monovalent H1N1 CA and H5N1 VN vaccines. **(B)** Densitometry analysis of positive HA2 bands in immunoblot **(A)**. **(C)** Immunoblot with H1N1 NC IWV and recombinant HAs of H1N1 CA, H2N2 SG, and H5N1 VN. The recombinant HAs were first treated with trypsin to generate HA2. **(D)** Densitometry analysis of positive HA2 bands in immunoblot **(C)**. Open arrowheads indicate HA2.

The results showed that the antibodies induced by the treated antigen exhibited a much higher reaction (≥2-fold) with HA2 not only from H1N1 NC IWV and H1N1 CA vaccine but also from H5N1 VN vaccine as compared to those by the untreated antigen (Figures 7A,B). These results were further confirmed with recombinant HA antigens. The recombinant HAs were first treated with trypsin to generate HA2 (28) before the immunoblot analysis. The results showed that the treated antigen also increased the antibody reaction with HA2 of all three recombinant HAs tested (H1N1 CA, H2N2 SG, and H5N1 VN) as compared to the untreated antigen (Figures 7C,D). This is consistent with the fact that H1, H2, and H5 subtypes are all in the same phylogenetic clade. These results together suggest that the enhanced antibody response against HA2 induced by antigens treated at mild low pH conditions might possibly be cross-protective against viruses from closely related subtypes.

## Discussion

Currently available inactivated influenza vaccines are poorly effective against variant or mismatched viruses. The studies described here suggest a potential highly practical approach to improve these vaccines by increasing their ability to cross protect against mismatched viruses. This approach is based on the finding that antigens treated at mild low pH conditions can induce a higher level of antibody response against the more conserved HA2 and enhance cross protection against challenge by heterologous or mismatched viruses.

**Figure 8:**
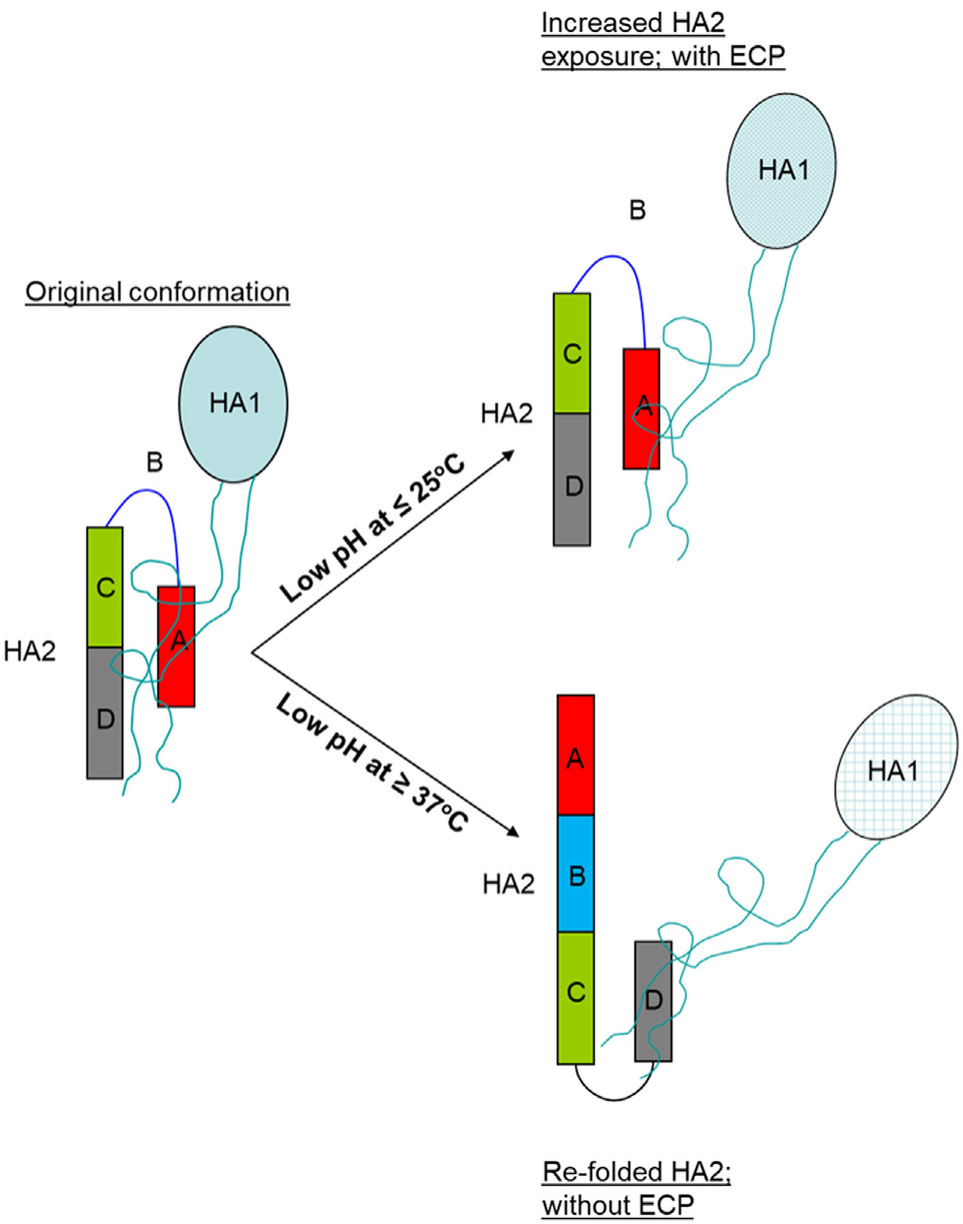
Schematic representation of HA structural changes following treatment at the mild low pH condition (≤25°C) and strong low pH condition (≥37°C). Abbreviations: ECP, enhanced cross protection; HA, hemagglutinin.

The HA is one of the best studied viral membrane proteins. Exposure to the low pH environment within endosomes (~pH 5.0, 37°C) causes drastic conformational changes in the HA which are required for membrane fusion during viral cell entry (10, 12, 29). These changes include the disassociation of HA1 and refolding and rising of HA2, such that it bears little conformational resemblance to its native or original structure. Thus, antibodies generated against such changed antigens are likely not or less protective against virus bearing native HA as it is only the immune response against the native structure that could be highly protective. This is consistent with the observation that antigens treated at such strong condition (~pH 5.0 and 37°C) are incapable of generating any enhanced cross protection (ECP) even though the refolded HA2 is fully exposed as shown in the present studies as well as by others (24). By contrast, treatment at mild low pH conditions (low pH and ≤25°C) likely caused only moderate structural changes which include increased exposure of HA2, but in its more native conformation without refolding. This is supported by moderately increased reaction with anti-CD antibody and EM showing that the HA spike, although less well defined, remained upright. Thus, the resulting enhanced antibody response against HA2 could be reactive with native antigens and therefore protective. This suggested mechanism for the ECP by antigens treated at mild low pH conditions is schematically presented in Figure 8.

The enhanced antibody response against HA2 by antigens treated at mild low pH conditions was clearly demonstrated by immunoblot in the present studies. This suggests that the linear epitopes on HA2 are likely involved. Antibodies specific to linear epitopes on HA can be highly protective as shown with monoclonal antibodies (30). Cross reactivity based on such epitope is likely more closely associated with amino acid sequence homology. On the other hand, no major increase in cross neutralization was induced by these antigens. Thus, these anti-HA2 antibodies are likely not strongly neutralizing and could mainly confer the ECP through ADCC. This is consistent with previous studies showing that non-neutralizing antibodies against HA stem are capable of protection against influenza *via* ADCC (15–17). In addition, inhibition of virus release could be another mechanism of protection by such antibodies (31).

It appears that enhanced cross-reaction and protection could be obtained at most mild low pH conditions including those causing no apparent loss of potency. However, the largest increase in cross protection was obtained under the conditions yielding a potency loss of ~50%. This was observed with both H1N1 and H3N2 antigens. The treated antigens with a ~50% potency loss remained capable of generating a high HAI titer with no or ≤2-fold reduction as compared to the untreated antigens. The specific condition (pH and temperature) used to obtain the optimal antigenic and structural changes can be readily identified for a given virus strain by a simple screening of the treatment conditions. This can be facilitated by the anti-CD ELISA which yields results in direct correlation with SRD and is simpler and quicker to perform. It could also be performed using on-plate low pH treatment of coated antigens. As the CD or HA2 is well conserved within a subtype, the polyclonal antibody specific to CD domain of subtype consensus sequence can cross-react well with individual strains within the subtype as shown in the present study, making it more advantageous than a monoclonal antibody for such analysis. This anti-CD ELISA could potentially also lend itself for evaluation of inactivated vaccines and individual viruses for HA structural integrity and acid sensitivity.

The antigens treated under the mild low pH conditions could therefore be used in an improved influenza vaccine with ECP. They could readily be formulated as the final vaccine to meet the potency standard with incorporation of additional treated or untreated antigens as necessary. The use of any additional antigens is likely well within the safety margin as there are already licensed high-dose inactivated influenza vaccines which contain four times the amount of the antigen used in the regular vaccines. It could also be formulated with an adjuvant without need for any additional antigen. For current inactivated vaccines, their potency (≥15 μg HA for each strain) as measured by SRD is correlated with the strain-specific HAI antibody titer and protection. A HAI titer of 1:40 is considered protective in humans. Thus, the improved vaccine with antigens treated at mild low pH conditions may not only provide strain-specific protection (the original vaccine indication) but also ECP. This improvement of the vaccine effectiveness could translate into significant reduction in influenza disease burden which could amount up to 35 million cases annually in the US alone (32, 33). This could be particularly true in light of that the effectiveness of current inactivated vaccines could be very poor or as low as 19% in recent years due largely to appearance of mismatched viruses (3). Incorporation of a low pH treatment step into vaccine manufacturing involves only use of simple acid and base for pH adjustment which does not introduce any impurities and can be performed without any major change of the manufacturing process.

It is known that the HA1, where the receptor binding site is located, is primarily responsible for inducing strain-specific HAI antibodies. It is highly variable as compared to the more conserved HA2. Thus, the improved vaccine with antigens treated at mild low pH conditions takes advantage of a more balanced use of HA1 and HA2 by combining the strain-specific immune responses mediated by HA1 with cross-reactive immune responses provided by HA2. It is particularly well suited as a TIV or QIV since an even broader cross-reaction and protection could be obtained by incorporating treated antigens from both H1 and H3 subtypes. Such cross protection might possibly be extended to viruses from closely related subtypes.

## Ethics Statement

All animal studies were conducted with the approval of the Institutional Animal Use and Care Committee at Texas A&M University.

## Author Contributions

YN and JG designed experiments. JG, YN, and DT performed experiments. YN, JG, and IT reviewed and analyzed the results. YN prepared the manuscript. JG, DT, and IT reviewed the manuscript.

## Conflict of Interest Statement

The authors declare that the research was conducted in the absence of any commercial or financial relationships that could be construed as a potential conflict of interest.

## References

[1] Osterholm MT Kelley NS Sommer A Belongia EA. Efficacy and effectiveness of influenza vaccines: a systematic review and meta-analysis. Lancet Infect Dis (2012) 12:36–44. 10.1016/S1473-3099(11)70295-X 22032844

[2] Zimmerman RK Nowalk MP Chung J Jackson ML Jackson LA Petrie JG 2014-2015 influenza vaccine effectiveness in the United States by vaccine type. Clin Infect Dis (2016) 63:1564–73. 10.1093/cid/ciw635 27702768 PMC5146719

[3] Centers for Disease Control and Prevention. Seasonal Influenza Vaccine Effectiveness, 2005-2017. Available from: https://www.cdc.gov/flu/professionals/vaccination/effectiveness-studies.htm (Accessed: February 28, 2018).

[4] Nabel GJ Fauci AS. Induction of unnatural immunity: prospects for a broadly protective universal influenza vaccine. Nat Med (2010) 16:1389–91. 10.1038/nm1210-1389 21135852

[5] Paules CI Marston HD Eisinger RW Baltimore D Fauci AS. The pathway to a universal influenza vaccine. Immunity (2017) 47:599–603. 10.1016/j.immuni.2017.09.007 29045889

[6] Erbelding EJ Post D Stemmy E Roberts PC Augustine AD Ferguson S A universal influenza vaccine: the strategic plan for the national institute of allergy and infectious diseases. J Infect Dis (2018) 218(3):347–54. 10.1093/infdis/jiy103 29506129 PMC6279170

[7] Fukuda K Levandowski RA Bridges CB Cox NJ Inactivated influenza vaccines. 4th ed In: Plotkin SA Orenstein WA Offit PA, editors. Vaccines. Philadelphia: Saunders (2003). p. 339–70.

[8] Wood J Schild G Newman R Seagroatt V Application of an improved single-radial immunodiffusion technique for the assay of haemagglutinin antigen content of whole virus and subunits influenza vaccines. Dev Biol Stand (1997) 39:193–200. 414951

[9] Fouchier RA Munster V Wallensten A Bestebroer TM Herfst S Smith D Characterization of a novel influenza A virus hemagglutinin subtype (H16) obtained from black-headed gulls. J Virol (2005) 79:2814–22. 10.1128/JVI.79.5.2814-2822.2005 15709000 PMC548452

[10] Bullough PA Hughson FM Skehel JJ Wiley DC. Structure of influenza haemagglutinin at the pH of membrane fusion. Nature (1994) 371:37–43. 10.1038/371037a0 8072525

[11] Carr CM Chaudhry C Kim PS. Influenza hemagglutinin is spring-loaded by a metastable native conformation. Proc Natl Acad Sci U S A (1997) 94:14306–13. 10.1073/pnas.94.26.14306 9405608 PMC24954

[12] Skehel JJ Wiley DC Receptor binding and membrane fusion in virus entry: the influenza HA. Annu Rev Biochem (2000) 69:531–69. 10.1146/annurev.biochem.69.1.531 10966468

[13] Chiu C Wrammert J Li GM McCausland M Wilson PC Ahmed R. Cross-reactive humoral responses to influenza and their implications for a universal vaccine. Ann N Y Acad Sci (2013) 1283:13–21. 10.1111/nyas.12012 23405860

[14] Corti D Suguitan AL Jr Pinna D Silacci C Fernandez-Rodriguez BM Vanzetta F Heterosubtypic neutralizing antibodies are produced by individuals immunized with a seasonal influenza vaccine. J Clin Invest (2010) 120:1663–73. 10.1172/JCI41902 20389023 PMC2860935

[15] Jegaskanda S Job ER Kramski M Laurie K Isitman G de Rose R Cross-reactive influenza-specific antibody-dependent cellular cytotoxicity antibodies in the absence of neutralizing antibodies. J Immunol (2013) 190:1837–48. 10.4049/jimmunol.1201574 23319732

[16] DiLillo DJ Tan GS Palese P Ravetch JV Broadly neutralizing hemagglutinin stalk-specific antibodies require FcγR interactions for protection against influenza virus in vivo. Nat Med (2014) 20:143–51. 10.1038/nm.3443 24412922 PMC3966466

[17] Vanderven HA Jegaskanda S Wheatley AK Kent SJ. Antibody-dependent cellular cytotoxicity and influenza virus. Curr Opin Virol (2017) 22:89–96. 10.1016/j.coviro.2016.12.002 28088123

[18] Krammer F Pica N Hai R Margine I Palese P. Chimeric hemagglutinin influenza virus vaccine constructs elicit broadly protective stalk-specific antibodies. J Virol (2013) 87:6542–50. 10.1128/JVI.00641-13 23576508 PMC3676110

[19] Steel J Lowen AC Wang TT Yondola M Gao Q Haye K Influenza virus vaccine based on the conserved hemagglutinin stalk domain. MBio (2010) 1(1):e00018–10. 10.1128/mBio.00018-10 20689752 PMC2912658

[20] Impagliazzo A Milder F Kuipers H Wagner MV Zhu X Hoffman RM A stable trimeric influenza hemagglutinin stem as a broadly protective immunogen. Science (2015) 349:1301–6. 10.1126/science.aac7263 26303961

[21] Yassine HM Boyington JC McTamney PM Wei CJ Kanekiyo M Kong WP Hemagglutinin-stem nanoparticles generate heterosubtypic influenza protection. Nat Med (2015) 21:1065–70. 10.1038/nm.3927 26301691

[22] Wang TT Tan GS Hai R Pica N Ngai L Ekiert DC Vaccination with a synthetic peptide from the influenza virus hemagglutinin provides protection against distinct viral subtypes. Proc Natl Acad Sci U S A (2010) 107:18979–84. 10.1073/pnas.1013387107 20956293 PMC2973924

[23] Ni Y Guo J Turner D Tizard I. Development of a novel dual-domain nanoparticle antigen construct for universal influenza vaccine. Vaccine (2017) 35:7026–32. 10.1016/j.vaccine.2017.10.051 29102171 PMC5715465

[24] Quan FS Li ZN Kim MC Yang D Compans RW Steinhauer DA Immunogenicity of low-pH treated whole viral influenza vaccine. Virology (2011) 417:196–202. 10.1016/j.virol.2011.05.014 21722934 PMC3152636

[25] Barrett T Inglis SC Growth, purification, and titration of influenza viruses. In: Mahy WJ, editor. Virology – A Practical Approach. Oxford: IRL Press (1985). p. 119–50.

[26] World Health Organization. WHO Technical Report Series, No. 927. Recommendations for the Production and Control of Influenza Vaccine (Inactivated). Geneva: World Health Organization (2005). Available from: http://www.who.int/biologicals/publications/trs/areas/vaccines/influenza/ANNEX%203%20InfluenzaP99-134.pdf?ua=1

[27] World Health Organization. WHO Manual on Animal Influenza Diagnosis and Surveillance. Geneva: World Health Organization (2002). Available from: http://www.who.int/iris/handle/10665/68026

[28] Wang K Holtz KM Anderson K Chubet R Mahmoud W Cox MM Expression and purification of an influenza hemagglutinin – one step closer to a recombinant protein-based influenza vaccine. Vaccine (2006) 24:2176–85. 10.1016/j.vaccine.2005.11.005 16310896 PMC7115642

[29] Di Lella S Herrmann A Mair CM. Modulation of the pH stability of influenza virus hemagglutinin: a host cell adaptation strategy. Biophys J (2016) 110:2293–301. 10.1016/j.bpj.2016.04.035 27276248 PMC4906160

[30] Tan GS Leon PE Albrecht RA Margine I Hirsh A Bahl J Broadly-reactive neutralizing and non-neutralizing antibodies directed against the H7 influenza virus hemagglutinin reveal divergent mechanisms of protection. PLoS Pathog (2016) 12(4):e1005578. 10.1371/journal.ppat.1005578 27081859 PMC4833315

[31] Yamayoshi S Uraki R Ito M Kiso M Nakatsu S Yasuhara A A broadly reactive human anti-hemagglutinin stem monoclonal antibody that inhibits influenza A virus particle release. EBioMedicine (2017) 17:182–91. 10.1016/j.ebiom.2017.03.007 28286060 PMC5360590

[32] World Health Organization. Influenza. Available from: http://www.who.int/immunization/topics/influenza/en/ (Accessed: July 26, 2017).

[33] Centers for Disease Control and Prevention. Disease Burden of Influenza. Available from: https://www.cdc.gov/flu/about/disease/burden.htm (Accessed: September 18, 2017).

